# Diabetes Mellitus and Liver Surgery: The Effect of Diabetes on Oxidative Stress and Inflammation

**DOI:** 10.1155/2018/2456579

**Published:** 2018-05-08

**Authors:** Mariana Mendes-Braz, Joilson O. Martins

**Affiliations:** Laboratory of Immunoendocrinology, Department of Clinical and Toxicological Analyses, School of Pharmaceutical Sciences of University Sao Paulo (FCF/USP), São Paulo, SP, Brazil

## Abstract

*Diabetes mellitus* (DM) is a metabolic disorder characterized by hyperglycaemia and high morbidity worldwide. The detrimental effects of hyperglycaemia include an increase in the oxidative stress (OS) response and an enhanced inflammatory response. DM compromises the ability of the liver to regenerate and is particularly associated with poor prognosis after ischaemia-reperfusion (I/R) injury. Considering the growing need for knowledge of the impact of DM on the liver following a surgical procedure, this review aims to present recent publications addressing the effects of DM (hyperglycaemia) on OS and the inflammatory process, which play an essential role in I/R injury and impaired hepatic regeneration after liver surgery.

## 1. Introduction

To extirpate a macroscopic lesion or accomplish a transplant, the blood flow to the liver must be interrupted to avoid the haemorrhagic process. Despite the safety of surgical procedures that involve the interruption of blood flow to the liver (ischaemia), this interruption contributes to tissue damage, which is potentiated by the restoration of blood flow (reperfusion). This phenomenon, known as ischaemia-reperfusion (I/R) injury [1, 2], is associated with inflammation and oxidative stress (OS) [3].

Diabetes mellitus (DM) is a metabolic disorder resulting from deficient insulin secretion and/or insulin action, leading to hyperglycaemia (high blood glucose) [4], which causes oxidative damage and activates inflammatory signalling cascades [5], in addition to acting as a damaging agent exacerbating the pathological conditions of DM [6, 7]. Considering the growing need for knowledge about the impact of DM on livers undergoing a surgical procedure, the present review aims to present recent data concerning the effects of DM (hyperglycaemia) on OS and the inflammatory process.

## 2. Oxidative Stress

Under normal conditions, the hepatic production of prooxidants, such as reactive oxygen species (ROS), is counterbalanced by antioxidants. An imbalance in favour of prooxidants corresponds to OS, and the direct action of ROS on cell viability and function is directly related to the occurrence of several pathological processes in the liver [8]. OS plays an essential role in liver surgery [9], and diabetes is generally followed by increased free radical production [10–13] or reduced antioxidant protection [14, 15]. To better understand the effect of DM (hyperglycaemia) on OS, this section will describe research findings that help clarify the association of DM with liver surgery.

### 2.1. Diabetes Mellitus and Ischaemia-Reperfusion Injury

**Figure 1:**
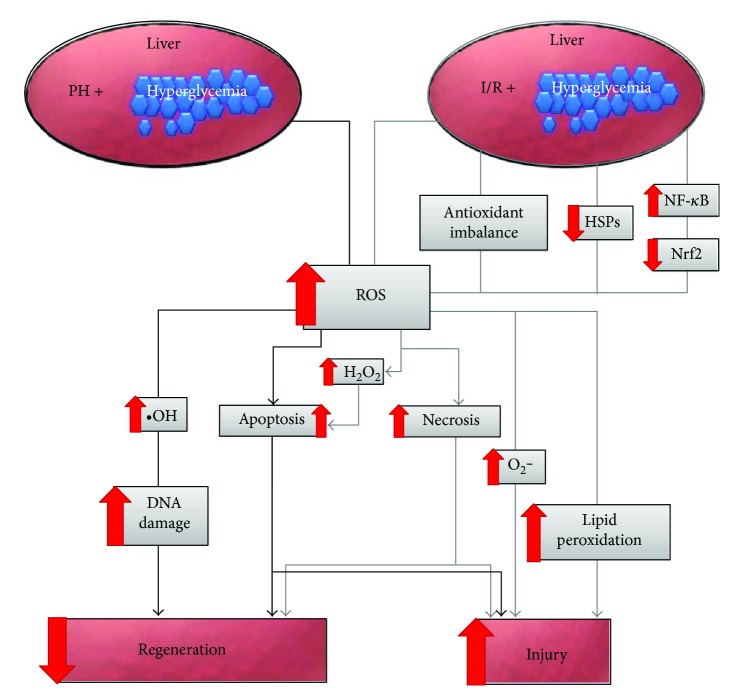
Mechanisms of OS in the promotion of liver damage and impaired regeneration after liver surgery in association with DM. The illustration shows the molecular events subsequent to the surgical procedure performed on the diabetic liver, which leads to a significant increase of ROS, inducing liver injury and regeneration. PH, partial hepatectomy; I/R, ischaemia-reperfusion; O_2_^−^, superoxide anion; HSP, heat shock protein; NF-*κ*B, nuclear factor kappa B; Nrf2, nuclear factor (erythroid-derived 2)-like-2 factor; H_2_O_2_, hydrogen peroxide; •OH, hydroxyl radical.

Hydrogen peroxide (H_2_O_2_), a mild and relatively stable oxidant that is formed in tissues exposed to I/R, has been considered a representative ROS for evaluating the response of cells to OS [16]. Although H_2_O_2_ is not a free radical, its accumulation may promote the formation of more toxic species, such as hydroxyl radicals (•OH), through the Fenton reaction [17]. H_2_O_2_ can cause permanent growth arrest [18, 19] and apoptosis [20–22] in a number of cell types. Nuclear (8-hydroxy-2′-deoxyguanosine) 8-OHdG formation indicates the presence of OS in nuclei [23]. The liver is a major organ affected by ROS [24] and is susceptible to the effects of OS induced by hyperglycaemia, causing liver injury [25–27]. Zhang et al. [28] found that serum H_2_O_2_ and nuclear 8-OHdG levels were higher in streptozotocin- (STZ-) induced diabetic rats subjected to I/R compared with the diabetic control group. ROS induce lipid peroxidation, which causes membrane injury, in addition to changes in ion permeability, enzyme activity, and, ultimately, cell death. Malondialdehyde (MDA), an indicator of oxidative injury produced via lipid peroxidation [29], is significantly enhanced in STZ-induced diabetic rats compared with normal rats and increases after I/R [28, 30] (Figure 1).

Apoptosis and necrosis can occur after I/R. An intense injury leads to initial necrotic killing, whereas late apoptosis may follow moderate injury [31]. STZ-induced diabetic rats exposed to an ischaemic period present significantly increased hepatocyte degeneration, sinusoidal dilatation, nuclear pyknosis, and cellular necrosis compared with the diabetes sham group [30]. In spite of this experimental difference, Behrends et al. [32] reported that necrosis is the preferential form of cell death in the liver of hyperglycemic rats (due to intraperitoneal injection of 25% glucose) subjected to I/R. The authors [32] noted that this increased injury may be associated with the inhibition of heat shock proteins (HSPs), which is only possible through the association of hyperglycaemia and I/R. The hyperglycaemia alone was not enough for HSP32 and HSP70 downregulation. HSPs are considered to be an indispensable protective agent against I/R injury because they are able to protect the liver from OS [33] (Figure 1).

Cell adaptation to OS is a consequence of the upregulation of distinct cytoprotective genes responsible for buffering the antioxidant capacity of the cell [34]. Under physiological conditions, an antioxidant defence system protects the body against the harmful effects of free radicals [35]. Diabetic livers are vulnerable to attack by oxygen free radicals because they present overall antioxidant depression [14]. Release of ROS and the concurrent consumption of endogenous antioxidants and cell death (apoptosis or necrosis) occur during hepatic I/R [36]. After I/R, nuclear factor (erythroid-derived 2)-like-2 factor (Nrf2), a transcription factor that mediates the expression of many endogenous antioxidants plays an important role in opposing hepatic injury [37]. Zhang et al. [28] reported that, after I/R injury, hepatocytes pretreated with high glucose (25 mM) exhibited a reduction in the antioxidative ability of the Nrf2 pathway and a substantial increase in nuclear factor kappa B (NF-*κ*B) translocation; however, NF-*κ*B activation was already enhanced in these hepatocytes before I/R injury. Interestingly, NF-*κ*B, a transcription factor that reacts to redox signals, may directly repress Nrf2 signalling at the transcriptional level [38, 39]. Zhang et al. [28] postulated that high glucose-induced ROS overproduction could initiate the inhibitory interaction between NF-*κ*B and Nrf2 (Figure 1**)**. However, the precise mechanisms involved in the NF-*κ*B and Nrf2 interaction under hyperglycaemic conditions require further elucidation.

Under normal conditions, the body presents a potent antioxidant system that is responsible for protecting it from the harmful effects of ROS [40]. Endogenous antioxidant enzymes attenuate I/R injury in the liver [36]. In both type 1 and type 2 DM, antioxidant defence enzymes are deficient, and there is an increase in oxidative damage [41]. High levels of ROS such as superoxide (O_2_^−^) are found in diabetes and especially during I/R injury [42]. Cem Sezen et al. [30] showed that there is an increase in glutathione s-transferase (GST) in STZ-induced diabetic mice post-I/R with respect to diabetic rats. Between these two groups, there was no difference in the level of superoxide dismutase (SOD); however, compared with the sham group (nondiabetic), there was a marked decrease in SOD levels. The orchestrated actions of several antioxidants in mammalian cells are essential for efficiently detoxifying free radicals. Therefore, any impairment in this pathway will influence the activities of other enzymes [43, 44]. Reduction in the activity of SOD will result in an increased level of O_2_^−^ [45]. GST is known to be an early and sensitive marker of liver injury and has been shown to increase after liver ischaemia/reperfusion [46]. This increased activity of GST could be explained as a compensatory mechanism to protect the organism against injury [47]. These findings are not only in accord with the diverse signalling pathways related to postoperative liver injury associated with DM (Figure 1**)** but also indicate the importance of the determination of increased ROS production and its characteristic consequences in postischaemic tissues, permitting the identification of interventions that stimulates ROS detoxification, and consequently protect against reperfusion injury [16], mainly in a diabetic context (Figure 1).

### 2.2. Diabetes Mellitus and Liver Regeneration

An increase in lipid peroxidation was found to be important for a normal proliferative process to occur in the liver remnant after partial hepatectomy (PH) [48, 49]. Francés et al. [50] reported that OS is increased by hyperglycaemia and is juxtaposed with the effect of PH in STZ-induced diabetic rats. Postoperative recovery depends on the regenerative capacity of the residual liver. The liver presents altered intracellular signalling pathways in type 1 DM specimens [51–53] and a consequent deficient regenerative response [54]. STZ-induced diabetic rats were found to present an increase in •OH production, which could result in DNA damage [55, 56] (Figure 1). Hyperglycaemia in STZ-induced diabetic rats leads to an increase in hepatic ROS production and is further enhanced after PH. STZ-induced diabetic rats subjected to PH present a decrease in the level of proliferating cell nuclear antigen (PCNA) and a significant decrease in cyclin D1 levels, suggesting that few hepatocytes are capable of entering the cell cycle [50].

Hyperglycaemia enhances •OH radical levels and consequent Bax protein induction. After PH, STZ-induced diabetic rats were found to present an increase in proapoptotic events (Bax/Bcl-xL ratio, caspase-3 activity, and cytosolic cytochrome c) compared with the diabetic group [50] (Figure 1**)**. The diversity of the results of different studies [30, 32, 50, 55] shows that the association of hyperglycaemia with different surgical modalities leads to differences in the type of cell death. It is imperative to identify the effects of diabetes on cell death after more complex surgical procedures leading to pronounced liver injury, such as liver transplantation and PH under I/R.

## 3. Inflammation

Hepatic inflammation is a complex process that is initiated in response to stressful conditions to protect hepatocytes from injury. However, overly intense inflammatory responses are followed by massive hepatocyte loss, causing irreversible parenchymal damage [57]. Liver damage is a serious complication in DM [58]. Surgical procedures induce acute inflammation, which is characterized by the production and release of various chemical mediators, including cytokines [59]. In the next section, the effects of DM (hyperglycaemia) on the hepatic inflammatory process after a surgical procedure will be discussed.

### 3.1. Diabetes Mellitus and Ischaemia-Reperfusion Injury

The pathophysiology of hepatic I/R injury is not only related to the direct cell impairment caused by ischaemic insult but also results from the restoration of blood flow, which triggers the proinflammatory environment. Diabetic patients present a variety of deficient immune cell functions [60, 61], and diabetic animals exhibit abnormalities in the course of the inflammatory response, with a consequent decrease in the number of leukocytes in inflammatory injuries [62, 63], the airway inflammatory response to antigen challenge [64, 65], mast cell degranulation [66, 67], superoxide generation, and tumour necrosis factor- (TNF-) *α* release by leukocytes upon exposure to lipopolysaccharides [68]. The difficulty in arriving at any consistent conclusion is due to the conflicting views regarding the impact of hyperglycaemia on inflammatory responses between different reports. Since clinical observations have revealed that the association between hyperglycaemia and immune alterations could increase the risk for rejection in transplantation, the substantial inflammatory response associated with I/R injury appears to be mediated by an exaggerated adhesion of leukocytes to the endothelium [69, 70].

**Figure 2:**
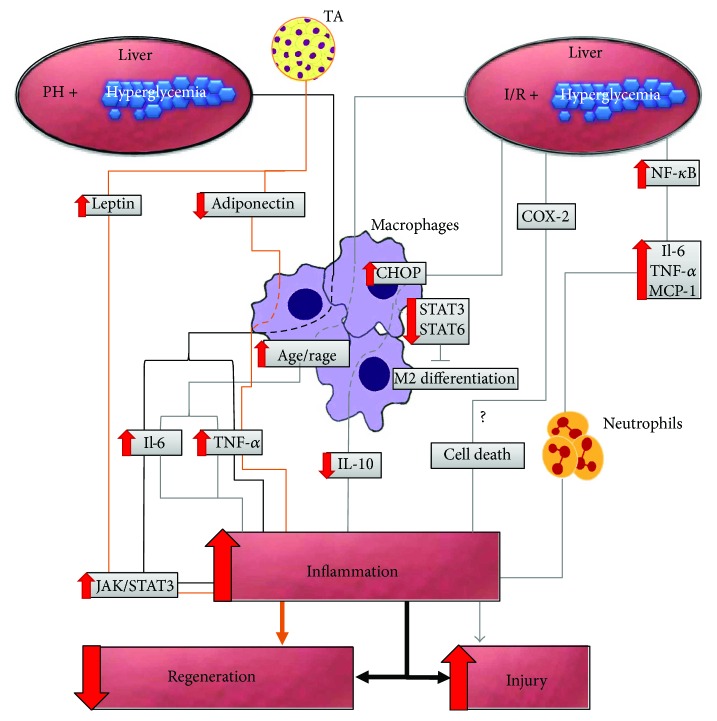
Inflammatory mechanisms underlying the promotion of liver damage and impaired regeneration after liver surgery in association with DM. The illustration shows the molecular events subsequent to the surgical procedure performed on the diabetic liver, inducing the participation of inflammatory cells and consequent cytokine production, leading to liver injury and regeneration. TA, adipose tissue; AGE, advanced glycation end products; RAGE, receptor for AGE; IL-6, interleukin-6; IL-10, interleukin-10; TNF-*α*, tumour necrosis factor-*α*; MCP-1, monocyte chemoattractant protein-1; JAK, Janus kinase; STAT3, signal transducer and activator of transcription 3; CHOP, C/EBP homologous protein; NF-*κ*B, nuclear factor kappa B; COX-2, cyclooxygenase-2; PH, partial hepatectomy; I/R, ischaemia-reperfusion.

The hyperinflammatory phenotype associated with DM may induce a liver immune response against I/R, which could favour an increase in parenchymal damage [71]. In the initial phase of liver injury, different events trigger a complex inflammatory pathway that leads to hepatic accumulation of neutrophils [72]. Through the release of oxidants and proteases, hepatocytes are directly damaged by recruited neutrophils, which are involved in by the later phase of liver injury induced by I/R [73]. In the livers of hyperglycaemic rats subjected to I/R, Behrends et al. [32] observed an increase in neutrophil infiltration (Figure 2**)**. Interestingly, in association with microvascular dysfunction in response to I/R, neutrophil infiltration is exacerbated in DM, suggesting that DM predisposes tissues to the detrimental consequences of I/R, which is a deleterious process that is broadly mediated by neutrophils [69].

The immune system responds to liver injury and/or stress through the activation of resident Kupffer cells (KCs), which release proinflammatory cytokines and other factors [74]. A prominent feature of liver injury is an increase in the hepatic macrophage population [75]. Considering cellular and molecular mechanisms, Yue et al. [71] showed that I/R stimulates the release of advanced glycation end products (AGE) into the blood of STZ-induced diabetic mice and that KCs express higher levels of the receptor for AGE (RAGE). The authors [71] proposed that RAGE may exhibit different functions in a cell type-specific manner. In normal mice, RAGE regulates hepatocyte proliferation during the restoration phase of I/R, whereas in diabetic mice, RAGE activates the hepatic immune system. These findings support the hypothesis that DM may be a factor involved in the course and evolution of I/R injury after liver surgery.

Activated KCs respond with a classic inflammatory reaction and consequent production of proinflammatory cytokines [76–80]. At 6 hours after reperfusion, TNF-*α* and interleukin- (IL-) 6 levels were found to be increased, while the IL-10 level was decreased on STZ-induced diabetic mice [71, 81] (Figure 2), whereas in control mice, KCs not only presented increases in TNF-*α* and IL-6 but also an increase in IL-10 [81]. The activation of IL-10 during a proinflammatory response may represent an important agent in the regulation of intensive inflammation in a stressful situation. These findings not only illustrate the defensive role of KCs during liver I/R injury in opposing the hyperinflammatory response through IL-10 expression but also show that hyperglycemic mice subjected to I/R present a significant decrease in IL-10 secretion, by KCs, which is related to uncontrolled inflammation and robust hepatic I/R injury [81].

Several studies suggest that endoplasmic reticulum stress and CHOP signalling could be upregulated by RAGE signalling [82–85]. After 6 hours of reperfusion, C/EBP homologous protein (CHOP) levels in KCs were found to be stimulated by I/R and were further increased in STZ-induced hyperglycemic mice. In hyperglycemic KCs, overactivation of CHOP is related to the inhibition of STAT3 and STAT6 activation. The signal transducers and activators of transcription (STATs) regulate the polarization of macrophages [86], and diabetic mice present M2 KC phenotype inhibition, which results in increased inflammation under hepatic I/R when the rodents exhibit interruption of IL-10-secreting M2 differentiation [81]. Additionally, mice that are only subjected to ischaemia show development of M2-type macrophages, which protect livers from I/R via an IL-10-dependent mechanism [87] **(**Figure 2**).**

In the pathogenesis of DM, activated innate immunity and inflammation are important factors. Type 2 DM involves inflammatory elements [88, 89], and type 1 DM is regarded as an inflammatory process [90]. NF-*κ*B is a transcription factor that is activated in the diabetic liver [91–93] and is involved in events that lead to inflammation [94]. NF-*κ*B regulates the expression of many inflammatory cytokines, including monocyte chemotactic protein- (MCP-) 1, IL-6, and TNF-*α* [95, 96], which are proinflammatory cytokines that may activate neutrophils and KCs [97]. Zhang et al. [28] showed that after 6 hours of reperfusion, the levels of these hepatic cytokines were significantly higher in STZ-induced diabetic rats and further increased after the ischaemic period. These results suggested that NF-*κ*B might also be involved in hepatic I/R in diabetic rats (Figure 2). The investigation of NF-*κ*B activation in diabetic livers subjected to surgical procedures should be extended to cell death. Between NF-*κ*B and TNF-*α*, there is an autocrine-reinforcing loop [98, 99]. The hepatic increase of TNF-*α* in STZ-induced diabetic rats leads to pronounced upregulation of the NF-*κ*B pathway [100], and NF-*κ*B activation induced by hyperglycaemia mediates cell apoptosis [101, 102].

Several inflammatory cytokines (e.g., TNF-*α*) and arachidonic acid metabolites (prostaglandins and thromboxanes) are involved in liver injury induced by I/R. Cyclooxygenase (COX) regulates the production of prostanoids [103], and inhibition of COX-2 protects against hepatic I/R injury [104, 105], which suggests that COX-2 is associated with organ injury and contributes to hepatic microvascular and hepatocellular injuries through TNF-*α* production [103]. Hepatocyte apoptosis stimulated by TNF is associated with c-Jun N-terminal kinase (JNK) activation [106]. Conversely, Francés et al. [107] showed that STZ-induced diabetic COX-2 transgenic mice presented a substantial decrease in apoptosis and that COX-2 overexpression could prevent the increase in JNK activity stimulated by high glucose. The authors [107] also showed that the increased expression of COX-2 in diabetic COX-2 transgenic mice induces an increase of phosphoinositide 3-kinase (PI3K) activity compared with diabetic wild-type mice, in addition to favouring the activation of Akt and producing an antiapoptotic signal [107]. These studies call attention not only to the contradictory roles of diabetes in orchestrating hepatocyte activity but also to the necessity of clearly understanding the consequences of diabetes for cell death after liver surgery (Figure 2).

### 3.2. Diabetes Mellitus and Liver Regeneration

In a model of type 2 DM (ob/ob murine), liver regeneration was found to be impaired after 70% PH, which resulted in 90% mortality [108]. The regenerative ability of the liver is compromised in type 1 diabetic rats subjected to PH [51, 52, 109]. In patients subjected to a major hepatectomy, DM tends to induce postoperative liver failure [110]. Considering the mechanisms of regeneration failure, diabetic and obese KK-Ay mice exhibit abnormal responses after PH [111] and present excessive induction of hepatic TNF-*α* expression. Although TNF-*α* is important for the initiation of normal hepatic regeneration [112, 113], excess induction of TNF-*α* in KCs might interfere with the regenerative process [111] (Figure 2).

Adipose tissue is involved in a number of biological functions, including inflammation, and acts as an endocrine organ through the secretion of several biologically active substances known as “adipokines” [114]. During liver regeneration, systemic adipose stores are required as a source of various adipokines, such as adiponectin, which is an essential signal for liver regeneration [115]. Aoyama et al. [111] showed that the serum adiponectin level was significantly reduced in KK-Ay mice before PH and tended to decrease gradually after PH. Adiponectin has been found to inhibit the lipopolysaccharide-dependent activation of macrophages [116, 117]. The significant hypoadiponectinemia presented by KK-Ay mice could be related to the fact that the KCs of these animals are more susceptible to certain stimuli; moreover, the hypoadiponectinemia caused by this susceptibility could be further associated with the increased production of TNF-*α* by KCs, which may interfere with regenerative responses [111] (Figure 2). Adiponectin mediates anti-inflammatory effects. However, since this role for adiponectin was found to depend on surgical conditions, the function of adiponectin in the inflammatory process is a controversial issue [118]. While injurious effects of adiponectin on steatotic livers subjected to warm ischaemia (60 minutes) were identified by Massip-Salcedo et al. [119], the beneficial (anti-inflammatory) effects of adiponectin on small fatty grafts subjected to cold ischaemia (40 minutes) were observed by Man et al. [120]. Although these findings were obtained in steatotic livers, these results suggest opportunities for investigation of the effect of adiponectin on diabetic livers subjected to different surgical procedures.

IL-6 is a protein synthesized by fibroblasts, monocytes, macrophages, T cells, and endothelial cells [121] that plays an important role in hepatic regeneration [122, 123]. Adipokines exhibit proinflammatory or anti-inflammatory activities [124], and leptin presents proinflammatory properties [125, 126]. IL-6 and leptin function in the Janus kinase- (JAK-) STAT3 signalling pathway [111]. KK-Ay mice present a substantial increase in the levels of IL-6 and leptin following PH [111]. Despite the important role of the JAK-STAT pathway in hepatic protection against different hepatic injuries [127, 128] and the evidence that IL-6, leptin, and the JAK-STAT signalling pathway are essential to liver regeneration [129–132], Aoyama et al. [111] showed that the role of the JAK-STAT pathway in hepatic regeneration seems to be complex and dependent on the intensity of the stimulus, showing that hyperphosphorylation of STAT3 favours poor hepatic regeneration as a result of direct downregulation of cyclin D1 expression (Figure 2).

## 4. Diabetes Mellitus in Clinical Situations

There is an absence of clinical studies elucidating signalling pathways related to liver damage and impaired regeneration in diabetic patients undergoing surgery. Nevertheless, it is indispensable to discuss and generate hypotheses about this issue, which is quite controversial because some studies have shown that DM patients present a poorer prognosis after hepatic surgery in comparison with non-DM patients, whereas others show no difference [133].

Focusing on the issues addressed in this review (OS and inflammation), Li et al. [133] and Shields et al. [134] described the typical change in microcirculation that occurs in diabetic patients after liver surgery. The ischaemic period and liver perfusion recovery are important factors related to hepatocellular damage because microcirculatory collapse is followed by a pronounced reduction of tissue oxygenation [135], which might result in degeneration and necrosis of hepatocytes and consequent liver dysfunction [136]. Experimental models of I/R injury have offered evidence that insufficient hepatic microcirculatory perfusion, inflammatory cell activation, and consequent generation of ROS, cytokines, and chemokines can be considered essential in I/R syndrome [137]. Although the authors [133, 134] did not report the relationship between diabetic liver failure after liver surgery and microcirculation collapse, we take this opportunity to raise this question for the development of future studies.

The alterations of hepatic haemodynamics are also related to hepatic steatosis, and a decrease in portal vein haemodynamics is observed in patients with a fatty liver disease [138, 139]. Moreover, experimental animals with steatosis present decreased parenchymal microcirculation [140]. Hepatic steatosis has long been reported in type 1 [141] and type 2 DM [142]. Steatosis is common in diabetic patients (36% incidence) [143], and increased steatosis raises the sensibility of the liver parenchyma to I/R injury [144]. In steatotic livers, the parenchymal regeneration ability is impaired, particularly after a surgical procedure [115], which may partially explain the incapacity of some diabetic patients to resist liver surgery. The high mortality observed in diabetic patients is absent in nondiabetic patients with steatosis [143]. In hepatocytes, increased accumulation of fatty acids induces OS arising from mitochondria, peroxisomes, or microsomes. ROS and lipid peroxidation products can influence KCs and stimulate NF-*κ*B activation, which in turn stimulates the production of TNF-*α* and several proinflammatory cytokines, such as IL-6 [143], which are presented in this review as factors involved in decreased regeneration and increased liver damage.

## 5. Conclusion

The purpose of this review was to discuss the literature addressing the damaging effect of DM on liver recovery after a surgical procedure and, especially, to highlight the need to expand knowledge of this issue to benefit patients with DM subjected to surgical procedures, which are increasing in clinical practice. Extensive work is still necessary to assess the differences between the diabetic and nondiabetic liver after a surgical procedure. Exploring this subject will enable the development of new treatments that will improve the success of diabetic liver recovery after surgery.
